# Cutaneous cytomegalovirus infection: a case report

**DOI:** 10.1093/skinhd/vzaf057

**Published:** 2026-04-22

**Authors:** Yunus Can Ozalp, Senem Maral, Sureyya Yigit Kaya, Huseyin Saffet Bekoz, Leylagul Kaynar

**Affiliations:** Faculty of Medicine, Istanbul Medipol University, Beykoz, Turkey; Faculty of Medicine, Istanbul Medipol University, Beykoz, Turkey; Faculty of Medicine, Istanbul Medipol University, Beykoz, Turkey; Faculty of Medicine, Istanbul Medipol University, Beykoz, Turkey; Faculty of Medicine, Istanbul Medipol University, Beykoz, Turkey

## Abstract

Cytomegalovirus (CMV) infection is common in people who are immunocompromised; however, cutaneous manifestations are rare compared with internal organ involvement. The clinical findings of cutaneous CMV infection typically include perioral or perianal ulcerations. Here, we present a case of a 66-year-old woman who underwent allogeneic haematopoietic stem cell transplantation, developed graft-versus-host disease and persistent skin lesions during her treatment course, and was eventually diagnosed with cutaneous CMV infection.

## Teaser

Cytomegalovirus (CMV) infection is common in immunocompromised patients, but cutaneous involvement is rare. We present a case of a 66-year-old female with graft-versus-host disease following allogeneic hematopoietic stem cell transplantation, who developed persistent skin lesions and was diagnosed with cutaneous CMV infection. This report highlights the clinical course, diagnostic challenges and successful treatment with intravenous ganciclovir, emphasizing the importance of early recognition in immunosuppressed patients.

What is already known about this topic?

- Cutaneous cytomegalovirus (CMV) infection is a rare manifestation that usually occurs in individuals who are immunocompromised.
- It may present with nonspecific ulcerative or necrotic skin lesions, which can mimic other infectious or inflammatory dermatoses.
- Diagnosis relies on histopathological examination and detection of CMV inclusion bodies or CMV DNA.

What does this study add?

- This case highlights the diagnostic challenges of cutaneous CMV infection in a patient who was immunocompromised.
- It emphasizes the importance of considering CMV in the differential diagnosis of atypical cutaneous ulcers.
- Early recognition and appropriate antiviral therapy can improve clinical outcomes.

Cytomegalovirus (CMV) infection is prevalent in people who are immunocompromised;^1^ however, cutaneous manifestations are uncommon compared with internal organ involvement.^2^ Clinical features of cutaneous CMV infection often include perioral or perianal ulcerations.^3^ Cutaneous CMV infection can present with bullous and ulcerative lesions, similar to those observed in bullous pemphigoid.^4^ In this case report, we describe a 66-year-old woman with a history of allogeneic haematopoietic stem cell transplantation (HSCT), graft-versus-host disease (GvHD) and multiple comorbidities who developed persistent skin lesions and was eventually diagnosed with cutaneous CMV infection.

## Case report

A 66-year-old woman diagnosed with myelodysplastic syndrome (MDS) had a bone marrow biopsy revealing a 7% increase in blasts. She underwent an allogeneic HSCT from her sibling on 28 January 2023, following a conditioning regimen with fludarabine and busulfan (HSCT risk score: 3). On day +6 post-HSCT, the patient reported left arm weakness, and cranial magnetic resonance imaging revealed infarcts. On day +9 post-HSCT, she experienced an epileptic seizure and was started on levetiracetam upon neurology consultation.

Despite ongoing treatment, the patient developed progressive dyspnoea and persistent fever, necessitating transfer to the intensive care unit (ICU) on day +13 post-HSCT. After stabilization, she was readmitted to the haematology ward on day +17 post-HSCT, for further supportive care.

Her past medical history included mitral valve insufficiency, hypertension, cerebrovascular disease, MDS, GvHD and cerebellar abscess. She had undergone lumbar hernia surgery (2022) and intracranial biopsy (2023), with no known drug or food allergies. Her regular medications included levetiracetam, dexamethasone, mirtazapine, amlodipine, ciclosporin and furosemide.

During her hospitalization, on day +44 post-HSCT, the first cutaneous manifestation was observed as an erythematous ulceration in the gluteal region. Dermatology consultation recommended topical treatment with an ointment containing 15 000 UI bacitracin and 150 mg neomycin sulfate, along with a zinc-based barrier cream. Despite continued treatment, by day +61 post-HSCT, the skin lesions had worsened, raising suspicion for acute cutaneous GvHD. A skin biopsy was performed for histopathological evaluation.

**Figure 1:**
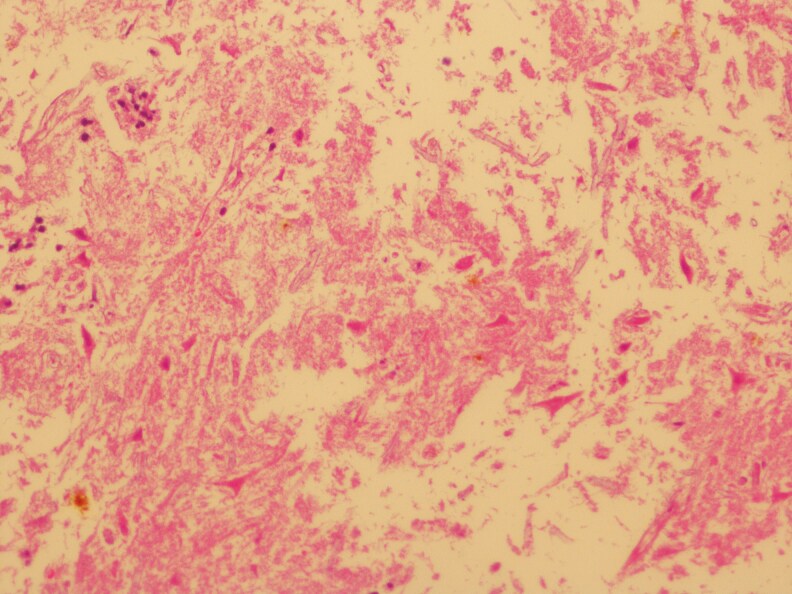
Histopathology image (haematoxylin and eosin, magnification × 100).

**Figure 2:**
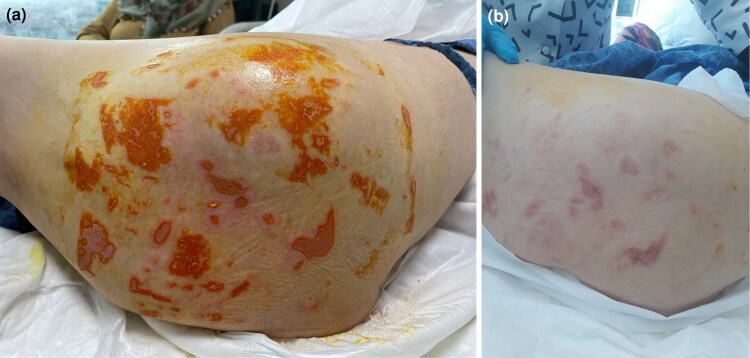
(a) Before treatment [active cytomegalovirus (CMV) ulcer]; (b) after treatment (healing CMV ulcer).

**Figure 3:**
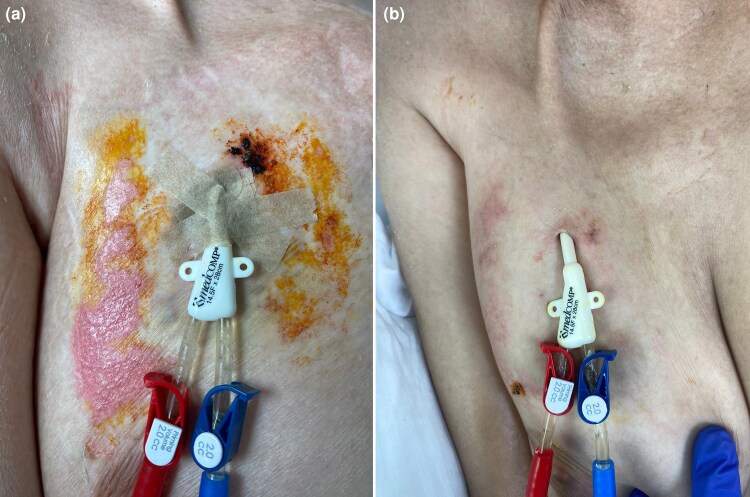
Cytomegalovirus ulcer around a catheter site: (a) pre- and (b) post-treatment comparison.

**Figure 4:**
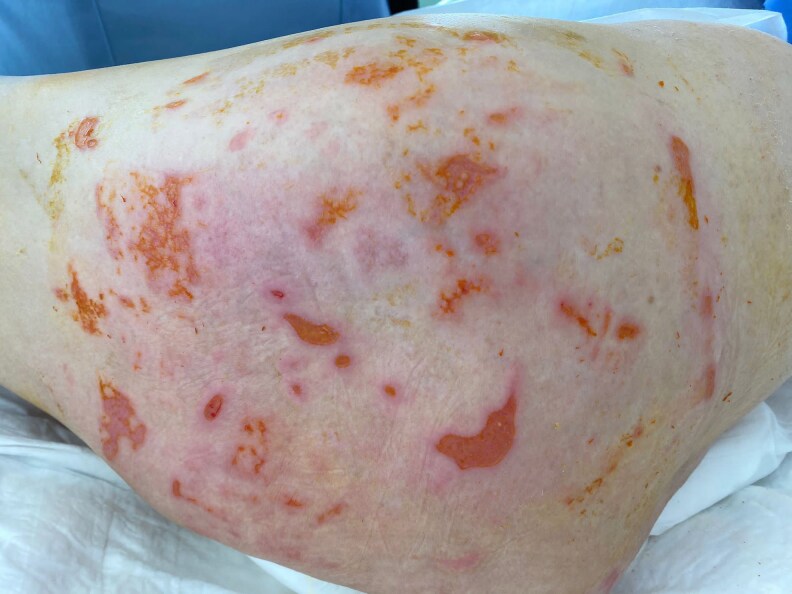
During treatment (healing phase initiation).

**Figure 5:**
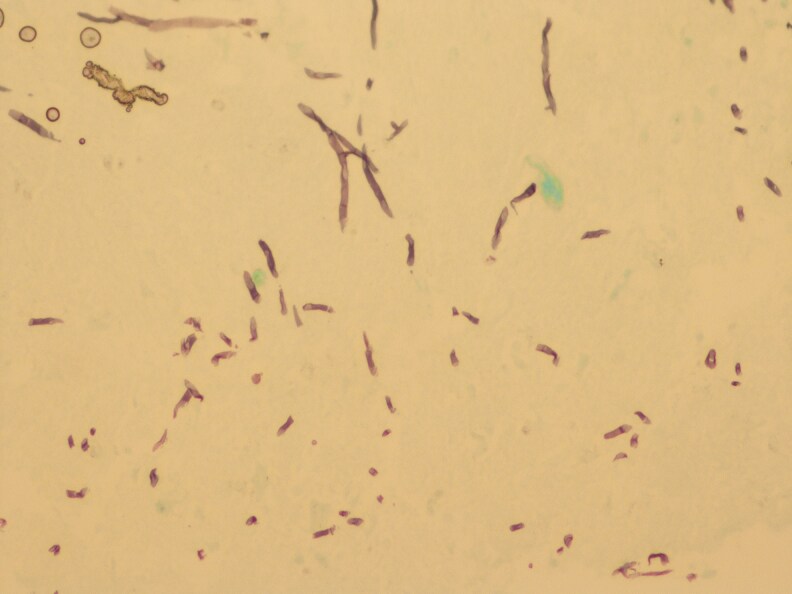
Periodic acid–Schiff (magnification × 100).

On day +65 post-HSCT, histopathological analysis ruled out acute cutaneous GvHD but confirmed CMV involvement of the skin. The dermatology department was reconsulted, noting the absence of purpuric, morbilliform, vesiculobullous eruptions or necrotic papules. They suggested serological testing for CMV (Figures 1–5).

On day +66 post-HSCT, quantitative CMV DNA polymerase chain reaction (PCR; plasma treated with ethylenediaminetetraacetic acid) revealed a high viral load of 50 688 copies mL^–1^. Infectious disease consultation recommended intravenous ganciclovir at a dose of 2 × 5 mg kg^–1^, which was initiated the same day. Subsequent CMV DNA PCR measurements showed a declining viral load: 11 498 copies mL^–1^ (11 April), 1338 copies mL^–1^ (14 April) and 212 copies mL^–1^ (20 April). On day +82 post-HSCT, CMV DNA became undetectable on PCR for the first time.

Following 18 days of antiviral therapy, intravenous ganciclovir was discontinued on day +84 post-HSCT. Further PCR tests on 25 April, 28 April and day +94 post-HSCT confirmed continued CMV negativity. Despite virological improvement, the patient’s overall condition deteriorated due to persistent comorbidities. On day +94 post-HSCT, she was transferred to the ICU due to respiratory failure and septic shock. Unfortunately, she developed cardiac arrest and was declared deceased on day 95 post-HSCT. Despite broad-spectrum antibiotic coverage, all cultures remained negative. Sepsis was considered to be of undetermined origin, likely due to bacterial translocation in the setting of profound immunosuppression.

## Discussion

CMV, also known as human herpesvirus 5 (HHV-5), is a member of the Herpesviridae family, possessing a large (220 nm) double-stranded DNA genome of 235 000 base pairs.^5,6^ The global seroprevalence of CMV in adults who are immunocompetent ranges from 40% to 100%.^7^ Approximately 80% of adults have CMV-specific antibodies.^8^ In individuals who are immunocompetent, CMV infection is often asymptomatic^9–12^ but may present as a mononucleosis-like syndrome.^13–18^ Immunosuppression can trigger viral reactivation and replication.^19–23^

Reports of cutaneous CMV infections in the literature are limited,^24–26^ possibly due to their rarity^27,28^ or challenges in histopathological diagnosis.^27,29–31^ Cutaneous CMV lesions may manifest as maculopapular rashes, urticarial eruptions, scarlatiniform erythema, cutaneous ulcerations, oral ulcerations, papules, nodules, morbilliform eruptions, verrucous lesions, perifollicular papulopustules or vesiculobullous eruptions.^32–34^ These lesions can be an early sign of disseminated CMV infection,^35,36^ which has been associated with an 85% mortality rate within 6 months.^37^

This case highlights a rare cutaneous manifestation of CMV in a patient who was immunocompromised and underscores the pivotal role of intravenous ganciclovir in managing cutaneous CMV infections. In patients with haematological malignancies, cutaneous lesions may indicate a severe systemic infection; therefore, prompt histopathological examination should be performed, particularly in patients who are immunosuppressed.

## Data Availability

The data underlying this article are included within the article. Further information is available from the corresponding author upon reasonable request.

## References

[1] Cohen JI, Corey GR. Cytomegalovirus infection in the normal host. Medicine 1985; 64:100–14. 2983175 10.1097/00005792-198503000-00003

[2] Choi YL, Kim JA, Jang KT et al Characteristics of cutaneous cytomegalovirus infection in non-acquired immune deficiency syndrome, immunocompromised patients. Br J Dermatol 2006; 155:977–82. 17034528 10.1111/j.1365-2133.2006.07456.x

[3] Baek G, Koo T, Kim MS, Jue MS. Cutaneous cytomegalovirus infection presenting as recalcitrant bullous pemphigoid lesion. Ann Dermatol 2023; 35:S97–9. 37853876 10.5021/ad.21.016 PMC10608396

[4] Casals DS, Nunes EdA, Maruta CW et al Disseminated cytomegalovirus disease as a cause of prolonged fever in a bullous pemphigoid patientunder systemic steroid therapy. J Dermatol 2003; 30:332–6. 12707471 10.1111/j.1346-8138.2003.tb00396.x

[5] Lambert EM . Cytomegalovirus ulcer. Arch Dermatol 2004; 140:1199. 15492181 10.1001/archderm.140.10.1199

[6] Fasanya AA, Pedersen FT, Alhassan S et al Cytomegalovirus cutaneous infection in an immunocompromised patient. Cureus 2016; 8:e598. 27335710 10.7759/cureus.598 PMC4895079

[7] Zuhair M, Smit GSA, Wallis G et al Estimation of the worldwide seroprevalence of cytomegalovirus: a systematic review and meta-analysis. Rev Med Virol 2019; 29:e2034. 30706584 10.1002/rmv.2034

[8] Kadambari S, Klenerman P, Pollard AJ. Why the elderly appear to be more severely affected by COVID-19: the potential role of immunosenescence and CMV. Rev Med Virol 2020; 30:e2144. 32671966 10.1002/rmv.2144 PMC7404358

[9] Bate SL, Dollard SC, Cannon MJ. Cytomegalovirus seroprevalence in the United States: the national health and nutrition examination surveys, 1988–2004. Clin Infect Dis 2010; 50:1439–47. 20426575 10.1086/652438 PMC11000537

[10] Stephanie ASS, Dollard SC, Radford KW et al Seroprevalence of cytomegalovirus infection in the United States. Clin Infect Dis 2006; 43:1143–51. 17029132 10.1086/508173

[11] Nolan N, Halai UA, Regunath H et al Primary cytomegalovirus infection in immunocompetent adults in the United States – a case series. IDCases 2017; 10:123–6. 29159070 10.1016/j.idcr.2017.10.008 PMC5684088

[12] Nangle S, Mitra S, Roskos S, Havlichek D. Cytomegalovirus infection in immunocompetent adults: is observation still the best strategy? IDCases 2018; 1:14. 10.1016/j.idcr.2018.e00442 PMC6129681 30202727

[13] Wang X, Yang K, Wei C et al Coinfection with EBV/CMV and other respiratory agents in children with suspected infectious mononucleosis. Virol J 2010; 7:247. 20858235 10.1186/1743-422X-7-247 PMC2949848

[14] Ziemann M, Thiele T. Transfusion-transmitted CMV infection – current knowledge and future perspectives. Transfus Med 2017; 27:238–48. 28643867 10.1111/tme.12437

[15] Jückstock J, Rothenburger M, Friese K, Traunmüller F. Passive immunization against congenital cytomegalovirus infection: current state of knowledge. Pharmacology 2015; 95:209–17. 25924667 10.1159/000381626

[16] Horwitz CA, Henle W, Henle G et al Clinical and laboratory evaluation of cytomegalovirus-induced mononucleosis in previously healthy individuals. Medicine (Baltimore) 1986; 65:124. 3005799 10.1097/00005792-198603000-00005

[17] Kiemola E, Von Essen R, Henle G, Henle W. Infectious-mononucleosis-like disease with negative heterophil agglutination test. Clinical features in relation to Epstein–Barr virus and cytomegalovirus antibodies. J Infect Dis 1970; 121:608–14. 4316146 10.1093/infdis/121.6.608

[18] Zhang Q, Gao Y, Peng Y et al Epidemiological survey of human cytomegalovirus antibody levels in children from Southeastern China. Virol J 2014; 11:123. 24996226 10.1186/1743-422X-11-123 PMC4094890

[19] Traylen CM, Patel HR, Fondaw W et al Virus reactivation: a panoramic view in human infections. Future Virol 2011; 6:451–63. 21799704 10.2217/fvl.11.21 PMC3142679

[20] Rouse BT, Sehrawat S. Immunity and immunopathology to viruses: what decides the outcome? Nat Rev Immunol 2010; 10:514–26. 20577268 10.1038/nri2802 PMC3899649

[21] White DW, Beard RS, Barton ES. Immune modulation during latent herpesvirus infection. Immunol Rev 2012; 245:189–208. 22168421 10.1111/j.1600-065X.2011.01074.x PMC3243940

[22] Virgin HW, Wherry EJ, Ahmed R. Redefining chronic viral infection. Cell 2009; 138:30–50. 19596234 10.1016/j.cell.2009.06.036

[23] Yu W, Geng S, Suo Y et al Critical role of regulatory T cells in the latency and stress-induced reactivation of HSV-1. Cell Rep 2018; 25:2379–89.e3. 30485807 10.1016/j.celrep.2018.10.105

[24] Drozd B, Andriescu E, Suárez A et al Cutaneous cytomegalovirus manifestations, diagnosis, and treatment: a review. Dermatol Online J 2019; 25:13030. 30710895

[25] Hajihashemi Z, Bidari-zerehpoosh F, Zahedi K et al Cytomegalovirus-induced cutaneous ulcer mimicking vasculitis in a patient with systemic lupus erythematous: a case report and review of the literature. Lupus 2021; 30:149–54. 33012246 10.1177/0961203320961473

[26] Arslan F, Batirel A, Mert A, Ozer S. Cytomegalovirus (CMV)-related cutaneous necrotizing vasculitis: case report and literature review. Braz J Infect Dis 2012; 16:482–5. 22975173 10.1016/j.bjid.2012.08.002

[27] Tuong W, Fazel N, Eisen DB. Factors influencing applicants’ ranking of dermatology residency programs in the national resident matching program. JAMA Dermatol 2015; 151:1378–80. 26502316 10.1001/jamadermatol.2015.3363

[28] Deerwester M, Rothman LR, Brinster NK. Pustulonodular cytomegalovirus infection. Hum Pathol Case Rep 2020; 22:337–42.

[29] Jahan M . Laboratory diagnosis of CMV infection: a review. Bangladesh J Med Microbiol 2010; 4:39–44.

[30] D’Alessandro M, Buoncompagni A, Minoia F et al Cytomegalovirus-related necrotising vasculitis mimicking Henoch–Schönlein syndrome. Clin Exp Rheumatol 2014; 32:73–5. 24854375

[31] Mégarbane B, Résière D, Ferrand J et al Difficulties in assessing cytomegalovirus-associated gastric perforation in an HIV-infected patient. BMC Infect Dis 2005; 5:28. 15829006 10.1186/1471-2334-5-28 PMC1087842

[32] Drago F, Aragone MG, Lugani C, Rebora A. Cytomegalovirus infection in normal and immunocompromised humans. A review. Dermatology 2000; 200:285–6. 10828625 10.1159/000018381

[33] Pariser RJ . Histologically specific skin lesions in disseminated cytomegalovirus infection. J Am Acad Dermatol 1983; 9:937–46. 6315790 10.1016/s0190-9622(83)70212-4

[34] Buckner FS, Pomeroy C. Cytomegalovirus disease of the gastrointestinal tract in patients without AIDS. Clin Infect Dis 1993; 17:644–56. 8268345 10.1093/clinids/17.4.644

[35] Feldman PS, Walker AN, Baker R. Cutaneous lesions heralding disseminated cytomegalovirus infection. J Am Acad Dermatol 1982; 7:545–8. 6292265 10.1016/s0190-9622(82)80255-7

[36] Mena Lora A, Khine J, Khosrodad N, Yeldandi V. Unusual manifestations of acute cytomegalovirus infection in solid organ transplant hosts: a report of two cases. Case Rep Transplant 2017; 2017:1–4. 10.1155/2017/4916973 PMC5611867 29085699

[37] Lee JY-Y . Cytomegalovirus infection involving the skin in immunocompromised hosts: a clinicopathologic study. Am J Clin Pathol 1989; 92:96–100. 2546421 10.1093/ajcp/92.1.96

